# A Rare Case Report of Extra-adrenal Pheochromocytoma Masquerading as a Pancreatic Head Malignancy

**DOI:** 10.7759/cureus.76464

**Published:** 2024-12-27

**Authors:** Bola Habeb, Sandy Khair, John Retzloff, Matthew Fowler

**Affiliations:** 1 Internal Medicine, University of Florida College of Medicine, Pensacola, USA; 2 Internal Medicine, Ascension Sacred Heart, Pensacola, USA; 3 Radiation Oncology, Cairo University, Cairo, EGY; 4 Radiation Oncology, National Cancer Institute, Cairo, EGY

**Keywords:** catecholamine-secreting tumour, extra-adrenal paraganglioma, hereditary paraganglioma-pheochromocytoma syndromes, neuroendocrine neoplasms, pancreatic head masses, pheochromocytoma differential, plasma metanephrine, retroperitoneal paraganglioma, urinary metanephrine

## Abstract

Extra-adrenal pheochromocytomas are rare neuroendocrine tumors originating outside the adrenal glands and can pose significant diagnostic challenges due to their variable presentations. This report highlights a case of an extra-adrenal pheochromocytoma masquerading as a pancreatic head malignancy. We underscore the importance of considering extra-adrenal pheochromocytoma in the differential diagnosis of pancreatic masses, particularly when biochemical or clinical features suggest catecholamine excess. Early recognition and appropriate management are crucial to avoid unnecessary interventions and improve patient outcomes.

## Introduction

An extra-adrenal pheochromocytoma, also known as paraganglioma, is a rare tumor arising from chromaffin cells outside the adrenal glands. Paragangliomas represent at least 10% of adult pheochromocytomas, occurring in approximately 1 to 4 cases per million people annually [1]. These tumors are similar to pheochromocytomas; however, they can arise from other organs along the sympathetic nervous system, most commonly below the diaphragm. Extra-adrenal pheochromocytomas can be associated with hereditary conditions such as multiple endocrine neoplasia (MEN) type 2, von Hippel-Lindau syndrome, or mutations in the *SDHB*, *SDHD*, or *RET* gene [2,3].

Patients commonly present with headaches, palpitations, diaphoresis, and hypertension due to excess catecholamine production. Diagnosis typically involves biochemical testing for elevated levels of catecholamines or their metabolites (i.e., metanephrines) in the blood or urine. Imaging techniques, such as CT scans, MRIs, or functional imaging (i.e., positron emission tomography (PET) scans), are used to locate the tumor. Surgical removal is the primary treatment. Preoperative management usually involves medications to control blood pressure and prevent intraoperative catecholamine crises. In cases of malignancy, additional treatments like chemotherapy, radiotherapy, or targeted therapies may be considered. Herein, we discuss a case of a retroperitoneal extra-adrenal pheochromocytoma masquerading as a pancreatic head tumor and emphasize the importance of thorough clinical correlation, including a detailed history, physical examination, and appropriate imaging studies, such as CT or MRI, to differentiate between the two conditions.

## Case presentation

We present a case of a 33-year-old African American female with a medical history significant for hypertension who presented to our facility for evaluation of headaches, heart racing, and uncontrolled blood pressure. Her symptoms started two weeks prior when she sought medical advice twice at the ED and was prescribed amlodipine and then lisinopril and hydrochlorothiazide for blood pressure control. Despite treatment, her symptoms persisted and progressively worsened to the extent that she developed heart racing, severe uncontrolled blood pressure, and repeated episodes of pre-syncope.

Vital signs on admission revealed elevated blood pressure of 210/120 mmHg, heart rate of 85 bpm, temperature of 37.3 °C, and oxygen saturation of 99% on ambient air.

Upon physical exam, the patient appeared anxious and distressed. The cardiovascular exam was normal, with a regular rate and rhythm and no audible murmurs. Pulmonary examination demonstrated clear lungs to auscultation with no wheezing, rales, or rhonchi. The abdominal exam revealed a soft, lax, and non-distended abdomen with audible bowel sounds in all quadrants.

Admission laboratory data is shown in Table 1.

**Table 1:** Admission laboratory data AST: aspartate aminotransferase; ALT: alanine aminotransferase; TSH: thyroid stimulating hormone

Parameters	Reference range, adults	Patient's values on admission
Hemoglobin (g/dL)	12.0–15.5	10.4
White cell count (per mm3)	3500–10500	13200
Platelet count (per mm3)	150,000–450,000	653000
Sodium (mEq/dL)	135–145	137
Potassium (mEq/dL)	3.5–5.1	3.7
Bicarbonate (mEq/dL)	22–29	22
Creatinine (mg/dL)	0.7–1.2	0.9
AST (units/L)	12–31	63
ALT (units/L)	9–29	52
Hep A IgM		Nonreactive
Hep Bs Ag		Nonreactive
Hep C Ab		Nonreactive
Hep B core IgM		Nonreactive
Beta hCG		Negative
TSH (mlU/L)	0.35-4.94	2.48
A1C	≤ 6.5	6.5

Imaging studies

**Figure 1:**
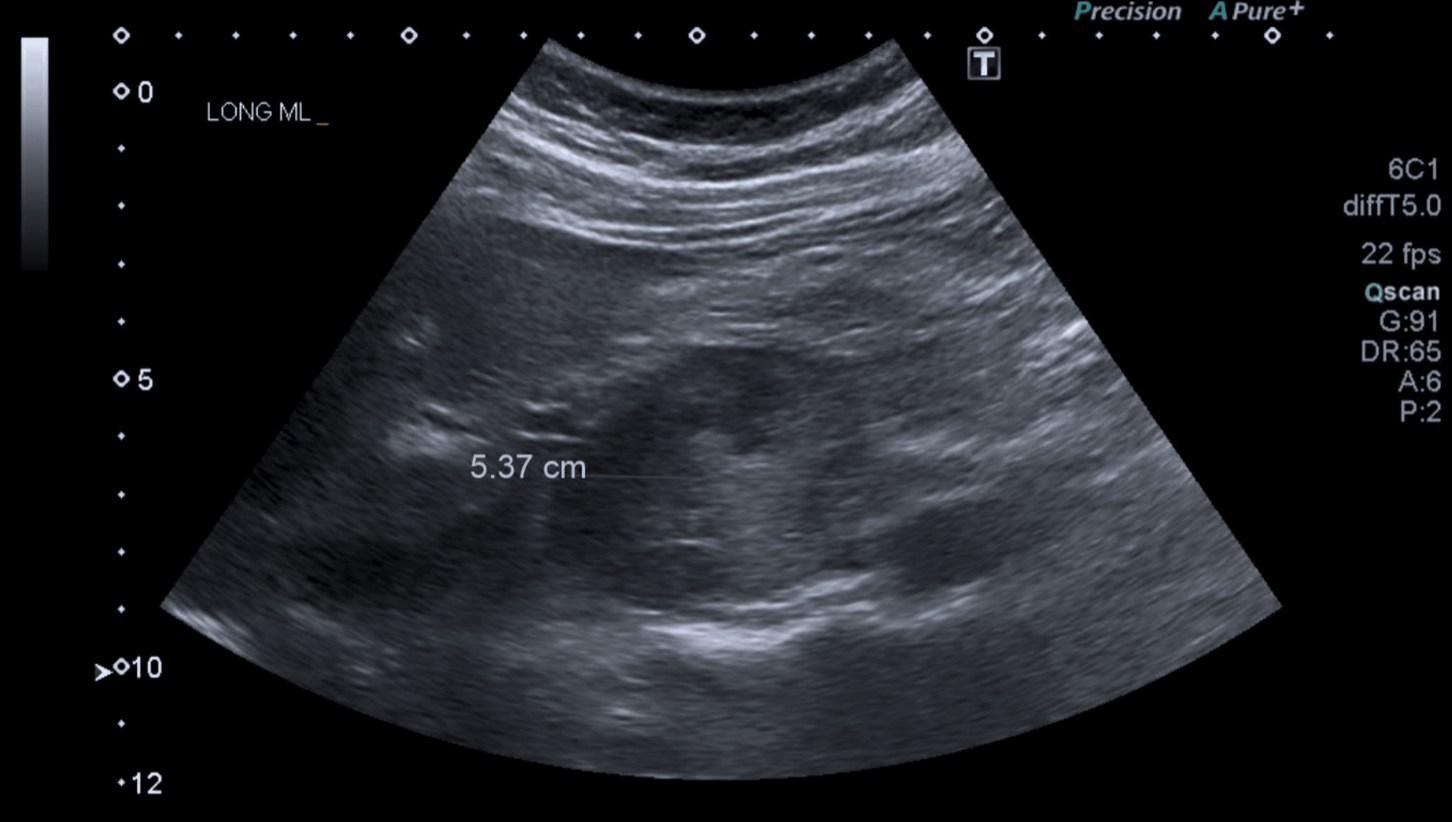
Abdominal ultrasound showing a pancreatic head mass

Abdominal ultrasound noted a 4.4 x 5.4 cm pancreatic head mass suspicious of malignancy (Figure 1).

**Figure 2:**
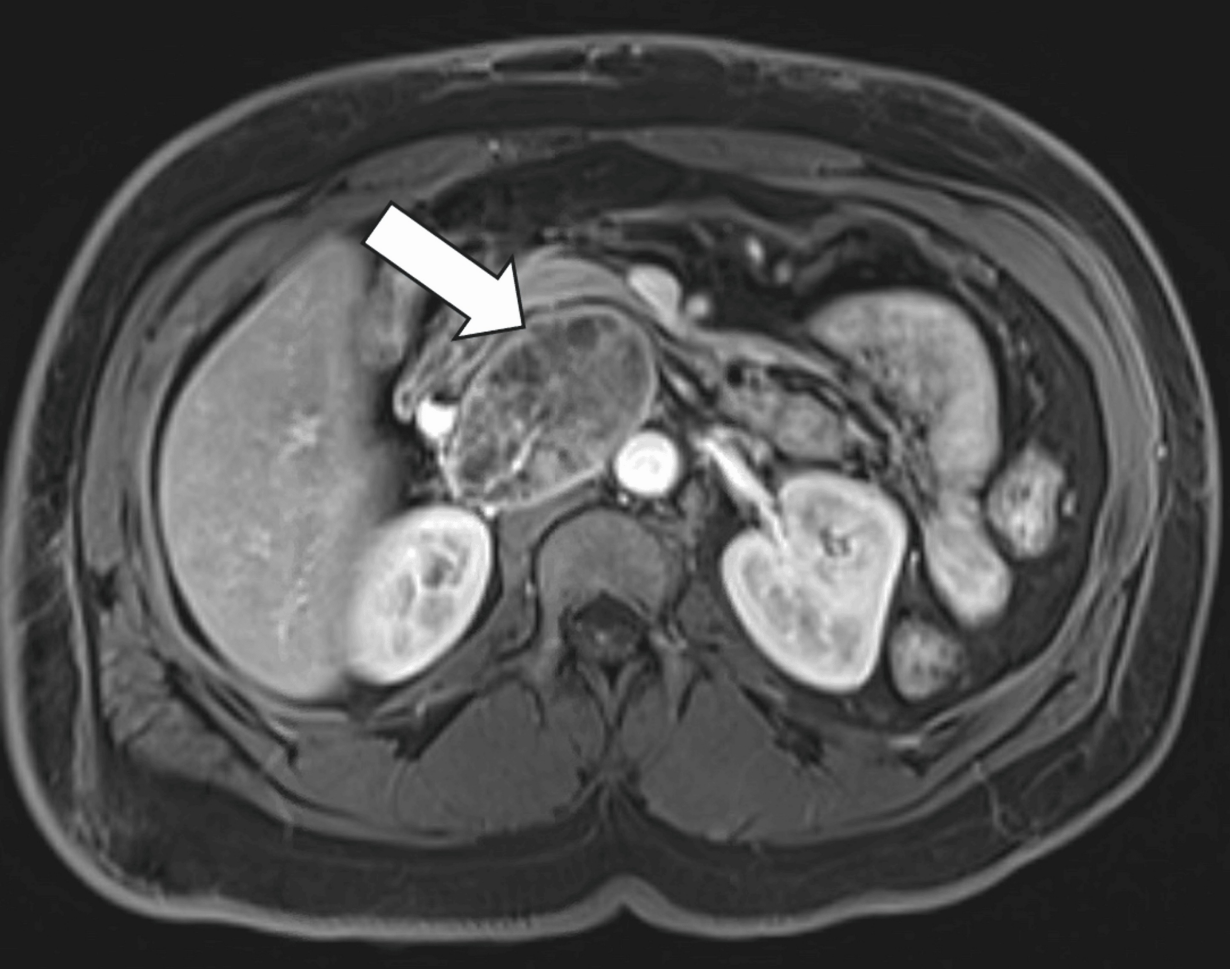
T2-HASTE (half-Fourier acquisition single-shot turbo spin-echo) MRI abdomen showing a 6.4 cm right retroperitoneum mass

**Figure 3:**
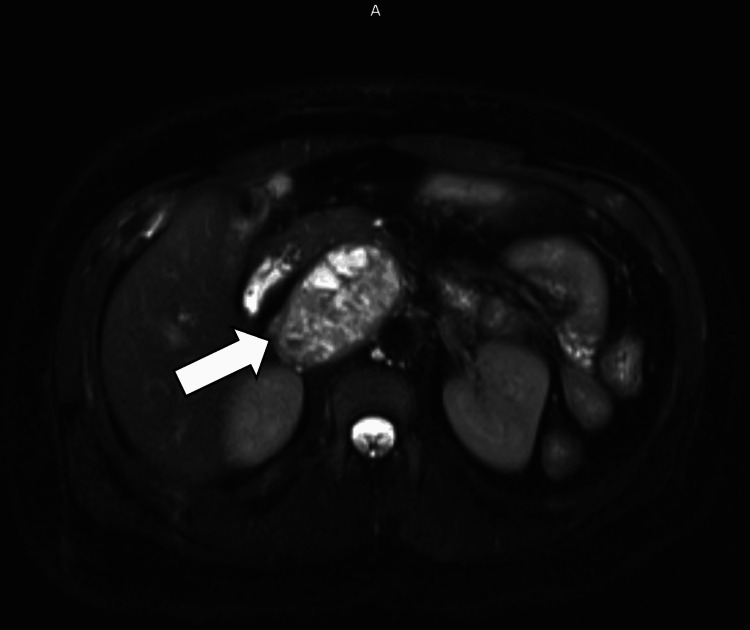
T2-HASTE (half-Fourier acquisition single-shot turbo spin-echo)-fast spin (fs) MRI abdomen showing a 6.4 cm right retroperitoneum mass

MRI of the abdomen with and without contrast showed a 6.4 cm mass in the right retroperitoneum, possibly of pancreatic origin with a solid pseudopapillary epithelial neoplasm (SPEN) of the pancreas or neuroendocrine tumors among the primary considerations (Figures 2-3).

A transthoracic echocardiogram showed a thickened left ventricle with hyperdynamic function and estimated EF > 70%. Bilateral renal Doppler ultrasound suggested renal artery stenosis due to compression by the retroperitoneal mass.

Hospital course

The patient’s blood pressure and heart rate fluctuations were highly suspicious for an extra-adrenal pheochromocytoma (i.e., paraganglioma) more than SPEN, and the patient was started on doxazosin for an alpha-adrenergic blockade and blood pressure control as well as propranolol for a beta-adrenergic blockade and heart rate control. A further workup revealed elevated plasma and urine metanephrines that came back one week later. Normal cancer antigen (CA) 19.9 levels ruled out pancreatic malignancy and normal morning and 24-hour urine cortisol levels ruled out adrenal pathology (Table 2).

**Table 2:** Further laboratory workup confirming pheochromocytoma CA: cancer antigen

Parameters	Reference range, adults	Patient’s values
Epinephrine level (pg/mL)	0.62	187
Norepinephrine level (pg/mL)	0.87	35886
Dopamine level (pg/mL)	0.48	301
Plasma normetanephrine (pg/mL)	0-210	7056
Plasma metanephrines (pg/mL)	0-88	268.7
Urine metanephrines (mcg/24h)	24-96	5322
CA 19-9 (u/mL)	≤ 37	< 2
Morning cortisol level (mcg/dL)	10-20	20.14
24-hour urine cortisol level (mcg/day)	46-131	64.4

The patient was evaluated by a multidisciplinary team consisting of endocrinology, hepatobiliary surgery, oncology, gastroenterology, and urology specialists. With medical therapy including alpha and beta-adrenergic blockade agents, the patient's general condition improved, her symptoms resolved, and her blood pressure and heart rate were better controlled. The patient was scheduled for outpatient resection of her extra-adrenal pheochromocytoma with recommendations to undergo a workup to rule out associated hereditary conditions such as MEN type 2, von Hippel-Lindau syndrome, and genetic testing for the exclusion of mutations in the *SDHB*, *SDHD*, or *RET* gene.

## Discussion

Pheochromocytomas are relatively rare neuroendocrine tumors arising from the adrenal medulla. The prevalence of pheochromocytomas ranges from 0.1% to 0.6%, with an incidence in the general population of 0.05% in an autopsy series. Pheochromocytomas have a slight predilection for females, with a female-male ratio of 1.4:1 [4]. An extra-adrenal pheochromocytoma, also known as paraganglioma, is a rare tumor arising from chromaffin cells outside the adrenal glands and represents 10% of adult pheochromocytomas. Approximately 25% to 35% of paragangliomas are often associated with hereditary conditions such as MEN type 2, von Hippel-Lindau syndrome, or mutations in the *SDHB*, *SDHD*, or *RET* gene [2,3].

Paragangliomas are further subclassified into secreting and non-secreting tumors. Symptoms and signs of paraganglioma are related to excess adrenaline or noradrenaline secretion into the blood; however, non-secreting paragangliomas are usually asymptomatic. Common symptoms of paraganglioma include episodes of headaches, palpitations, and diaphoresis. Less common symptoms are nausea, vomiting, constipation or diarrhea, and unexplained weight loss. These tumors can occasionally cause a life-threatening condition called a catecholamine crisis or hypertensive emergency, where blood pressure spikes dangerously, leading to potential heart attacks, strokes, or multiorgan failure.

The variable clinical presentations and biological behaviors of paragangliomas often make accurate diagnosis challenging. Asymptomatic cases can be found incidentally while ordering a test or procedure for another reason. Symptomatic patients should undergo a detailed medical history, including a previous pheochromocytoma or a family history of paraganglioma, a thorough physical exam, and a comprehensive medical evaluation. Diagnosis typically involves biochemical testing for elevated levels of catecholamines or their metabolites (metanephrines) in the blood or urine. Imaging techniques, such as CT scans, MRIs, MIBG (metaiodobenzylguanidine) scans, and PET scans, especially with tracers like 18F-DOPA or Gallium-68, are highly sensitive and specific for detecting paragangliomas and assessing metastasis [5].

Ultimately, surgery is the treatment of choice for these tumors, with a cure rate of up to 90% of cases. According to the literature, patients who undergo any surgery with undiagnosed underlying pheochromocytoma have an operative mortality rate of 5.7%. Fortunately with proper diagnosis, advances in medical imaging, and appropriate preoperative preparation, the current mortality rate is approaching 0% [6]. Preoperative preparation is crucial to reduce the risks of perioperative morbidity and mortality [7]. Alpha and beta blockade is a critical preoperative management strategy for patients with a pheochromocytoma to prevent complications like hypertensive crises during surgery. The primary goal of an alpha blockade is to control hypertension and prevent hypertensive crises during surgery by inhibiting vasoconstriction caused by catecholamines while beta blockers are added to control tachycardia and arrhythmias resulting from catecholamine excess or reflex tachycardia due to vasodilation from alpha blockade. Postoperative hypotension can occur in 20-70% of cases due to sudden catecholamine withdrawal, thus close monitoring of arterial pressure is mandatory after surgery [7].

With early detection and treatment, the prognosis for benign pheochromocytomas is excellent. For malignant or recurrent pheochromocytomas, long-term management and follow-up are required. Genetic counseling is often recommended for patients with hereditary forms.

## Conclusions

Extra-adrenal pheochromocytoma is a rare differential diagnosis in patients presenting with a retroperitoneal mass mimicking a pancreatic head tumor. However, with the described pitfalls, it is important to correlate with the clinical presentation and do an appropriate workup measuring catecholamines and their metabolites (e.g., plasma or urine metanephrines) for early identification and differentiation of pheochromocytomas from other tumors. Surgery is curative, but long-term surveillance and genetic counseling are also necessary to guide the patient in understanding their risks for familial tumors and inform their relatives about possible genetic testing.
